# Hepatitis B vaccine effectiveness among vaccinated children in Africa: a systematic review and meta-analysis

**DOI:** 10.1186/s12887-024-04557-w

**Published:** 2024-02-28

**Authors:** Mekuanint Geta, Endalew Yizengaw, Tsegahun Manyazewal

**Affiliations:** 1 Translational Medicine Program, Center for Innovative Drug Development and Therapeutic Trials for Africa (CDT-Africa), College of Health Sciences, Addis Ababa University, Addis Ababa, Ethiopia; 2 Department of Medical Microbiology, School of Laboratory Sciences, College of Medicine and Health Sciences, University of Gondar, Gondar, Ethiopia; 3 Department of Laboratory Science, College of Medicine and Health Sciences, Bahir Dar University, Bahir Dar, Ethiopia

**Keywords:** Africa, Anti-hepatitis B surface, Hepatitis B vaccine, Hepatitis B virus, Meta-analysis

## Abstract

**Background:**

Globally, 257 million people have chronic hepatitis. Even though a safe and effective prophylactic vaccine against HBV infection has been available, it causes significant morbidity and mortality. HBV vaccines were designed to improve or modulate the host immune responses. The effectiveness of the vaccine is determined by measuring serum hepatitis B surface antibody (Anti-HBs) level. Therefore, this systematic review aimed to evaluate the effectiveness of hepatitis B vaccine among vaccinated children.

**Methods:**

Preferred reporting items for systematic review and meta-analysis (PRISMA) guidelines was applied for systematically searching of different databases. Only cross-section studies measuring the level of anti-HBs of vaccinated children were included. The seroprotective level with anti-HBs > 10mIU/ml was extracted. The meta-analysis was performed using statistical software for data sciences (STATA) version 14. Effectiveness estimates were reported as a proportion of anti-HBs level. The heterogeneity between studies was evaluated using the I^2^ test, and I^2^ > 50% and/or *P* < 0.10 was considered significant heterogeneity. Significant publication bias was considered when Egger’s test P-value < 0.10. The new castle Ottawa scale was used to assess the quality of the studies.

**Results:**

A pooled sample size of the included papers for meta-analysis was 7430. The pooled prevalence of seroprotected children was 56.95%, with a heterogeneity index (I^2^) of 99.4% (*P* < 0.001). 35% of the participants were hypo-responders (10-99mIU/ml) and 21.46% were good responders (> 100mIU/ml). Based on subgroup analysis using country of studies conducted, the highest prevalence of anti-HBs was 87.00% (95% CI: 84.56, 89.44), in South Africa, and the lowest was 51.99% (95% CI: 20.41–83.58), with a heterogeneity index I^2^ = 70.7% (*p* = 0.009) in Ethiopia.

**Conclusion and recommendations:**

Hepatitis B vaccine seroprotective level in the current pooled analysis have suboptimal, which failed to demonstrate consistent effectiveness for global hepatitis B virus elimination plan in 2030. Using consistent age group may have a significant value for the decision of the HB vaccine effectiveness. A significant heterogeneity was observed both in studies conducted in Ethiopia and Egypt. Therefore, the impact of HB vaccination on the prevention of hepatitis B virus infection should be assessed regularly in those countries. Future meta-analysis is needed to investigate all possible vaccines in a separate way of reviewing, which will lead to a strong conclusion and recommendations.

**Supplementary Information:**

The online version contains supplementary material available at 10.1186/s12887-024-04557-w.

## Introduction

Hepatitis B virus (HBV) infection is a serious health problem causing a substantial burden of acute and chronic liver disease [1, 2]. Today, more than 257 million people worldwide are chronically infected with HBV. Despite an effective vaccine, the virus causes about 887,000 deaths yearly [3]. Of chronic hepatitis B (CHB), 61 million live in Africa [4, 5]. The development of CHB among newly infected persons depends on their age at the time of infection. More than 90% of infected infants, 25–50% under five years of age and 6–10% of acutely infected older children develop chronic infection [6, 7]. Up to 25% of infants and older children who acquire HBV infection develop HBV-related hepatocellular carcinoma (HCC) or cirrhosis [6, 7].

The World Health Organization (WHO) 2016 adopted a strategy to globally eliminate HBV infection as a public health threat by 2030, to reduce its incidence by 90% and its mortality by 65% [8, 9]. Studies in areas with high HBV endemicity have shown declines in the prevalence of chronic HBV among children to < 2% after routine infant immunization [10, 11].

Universal vaccination against HBV in newborns was found to be easier and cost-effective. Many countries have gradually adopted the HBV vaccine in their national immunization programs since the WHO recommended vaccination for children in the 1990s. As of 2007, 171 (89%) of the 193 WHO member states had initiated a hepatitis B vaccination program [11, 12]. Vaccination with hepatitis-B vaccine has been considered an important tool for protection against HBV infection [13].

An anti-HBs concentration of ≥ 10 mIU/mL measured 1–3 months after administering the last dose of the initial vaccination series is considered a reliable marker of protection against infection [14, 15]. The hepatitis-B vaccination should produce a protective level of anti-HBs in ≥ 95% of the vaccinated individuals after completion of the recommended vaccination schedule [14]. After primary hepatitis B immunization, anti-HBs concentrations decline rapidly within the first year and more slowly thereafter [16]. To our knowledge, no meta-analysis assesses the anti-HBs level among vaccinated children in Africa. Thus, this meta-analysis aimed to evaluate the evidence of the effectiveness of HB vaccine among vaccinated children in Africa.

## Methods

### Searching strategy

This systematic review was performed following preferred reporting items for systematic review and meta-analysis (PRISMA) guidelines to search scientific literature (Supplementary 1). We first searched published articles from PubMed, Medline and Google Scholar databases using the following keywords and medical subject headings (MeSH):

“Hepatitis B vaccine or HB vaccination,” AND “effectiveness or immune response”.

“Hepatitis B vaccine or HB vaccination,” AND “Antibody levels”.

“Hepatitis B vaccine or HB vaccination,” AND “African children”.

“Hepatitis B vaccine or HB vaccination,” AND “children or cross-sectional”.

“Hepatitis-B” AND “Vaccine or vaccination or immunization”.

Mekuanint Geta (MG) and Endalew Yizengaw (EY) searched PubMed and Medline and google scholar databases over a period of January to February 2022. Only peer-reviewed original articles published in English were searched. We also had searched from the reference lists of all searched articles to further search similar studies and references.

Population: children who took HBV vaccine.

Intervention: different types of hepatitis-B vaccine.

Comparator: None.

Outcome: The outcome of this systematic review was the percentage of vaccinated children with anti-HBs titer ≥ 10 mIU/ml.

Inclusion: Papers published in English language with a cross-sectional study design reporting the level of anti-HBs from vaccinated children.

Exclusion criteria: We excluded review papers or any other study with unreliable data or not reporting original data.

### Data extraction and quality assessment

The titles, abstracts and contents of the study were reviewed according to our inclusion and exclusion criteria. Two independent authors: MG and EY extracted the data using a data extraction format prepared in a Microsoft Excel 2019 spreadsheet. The data extracted were the first author’s name, publication year, Study population, country, study design, sample size, number of patients with anti-HBs titer (< 10mIU/ml, > 10mIU/ml, 10-99mIU/ml, > 100mIU/ml), vaccine type, and vaccination schedule. The quality of each study was assessed using the modified Newcastle-Ottawa Scale (NOS) [17], and evaluated independently by two authors (MG and EY). Any disagreements that appeared during abstraction were resolved by discussion and consensus. Any confusion regarding the results of the selected study was resolved through mutual agreement (Supplementary 2).

### Data analysis

Statistical software for data sciences (STATA) version 14 was used to carry out the meta-analysis. Proportions were estimated using a random effects model with a 95% confidence interval (CI). Subgroup analysis was performed to address heterogeneity. The country of the study conducted was used as the grouping variable.

The heterogeneity between studies was evaluated using the I^2^ test, which describes the percentage of variability in effect estimates because of heterogeneity beyond sampling error [18]. Heterogeneity across the studies was checked using the *I*^*2*^ tests, where *P* < 0.10 or I^2^ > 50% indicates significant heterogeneity. A sensitivity analysis was performed by removing one study at a time to confirm that any single study did not drive our findings. The statistical significance was defined as P-value < 0.05. Publication bias was checked with Egger’s regression test and represented graphically by a standard funnel plot when there were ten or more studies. Egger’s test with P-value < 0.10 was considered significant. In addition, the quality of studies was also performed using New Castel Ottawa scale for observational studies.

## Results

### Study characteristics

**Fig. 1:**
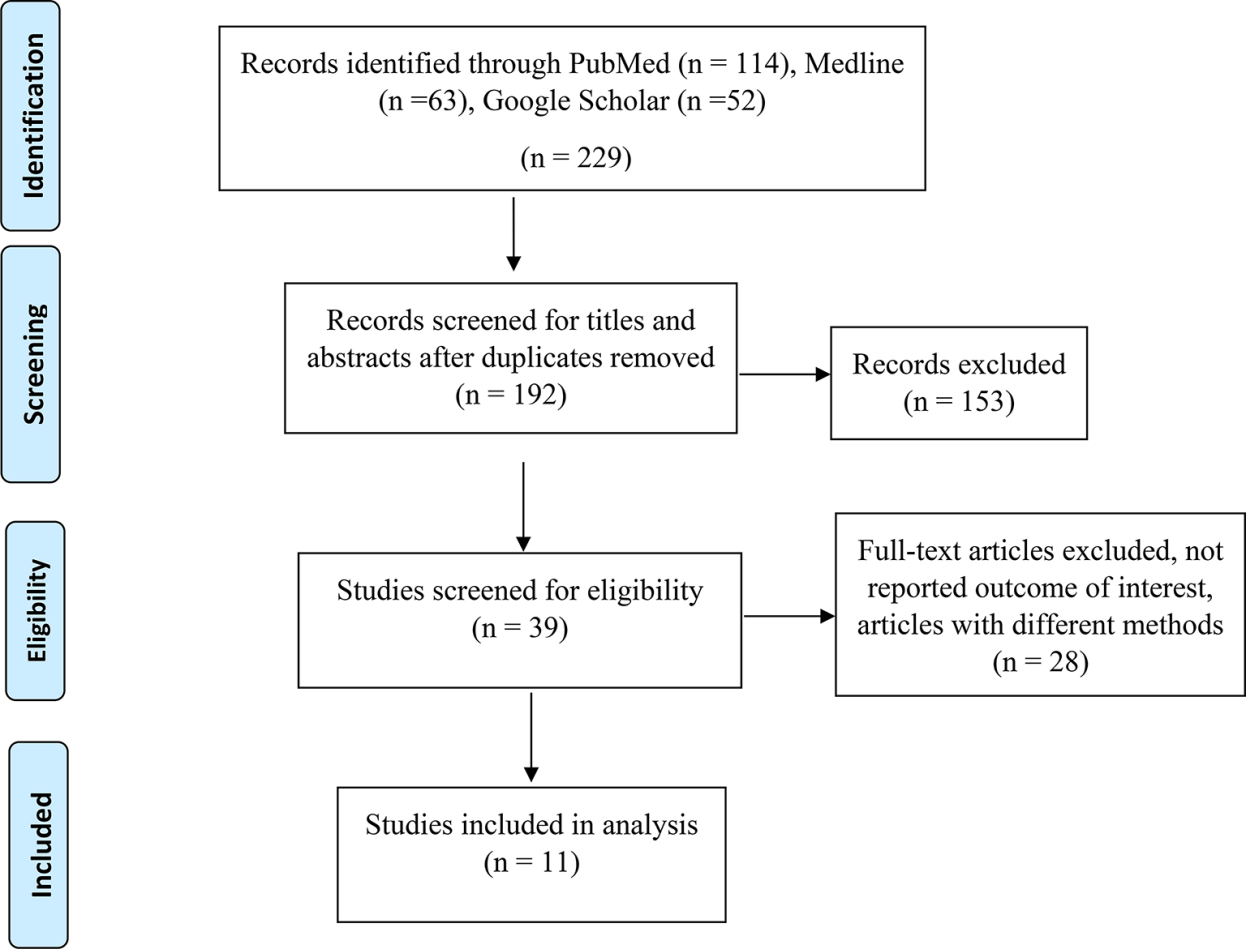
PRISMA flow diagram of screening and selection processes

Initially, we identified 192 unique records through different database searches after removing duplicates. The titles and abstracts were screened, and 153 papers were excluded. Thirty-nine articles were assessed for eligibility; 28 articles did not report the outcome of interest, and articles with different methods were excluded. Finally, 11 articles were included in the meta-analysis (Fig. 1).

A pooled sample size of the eleven articles included for meta-analysis was 7430. All the included studies were cross-sectional, and the sample size ranged from 170 [19] to 3600 [20]. Among those studies included in this review, seven reported hypo-responders and good responders separately. Regarding the country distribution of the included studies, five were conducted in Egypt [19–23], five in Ethiopia [24–28], and one in South Africa [29]. All the included studies had enrolled a full-dose hepatitis B vaccination coverage. A summary of the included studies, including the vaccine types, routes of administration, vaccination schedules and sample size were also provided in Table 1. All of the studies focused on children as the targeted population. The number of doses administered to children in different countries was three doses.

**Table 1:** The general characteristics of the studies included in this systematic review

Author (Year)	Country	Study Design	Age in Years	Population	Sample Size	Vaccine Type	Route	Vaccination Schedule
El-Melligy (2013)^21^	Egypt	Cross-sectional	2 to16	Children	189	rDNA	IM	2, 4, 6 mths
Salama (2015)^20^	Egypt	Cross-sectional	1 to 16	Children	3600	rDNA	IM	2, 4, 6 mths
Elrashidy (2013)^19^	Egypt	Cross-sectional	5 to 15	Children	170	rDNA	IM	2, 4, 6 mths
Teshome (2019)^24^	Ethiopia	Cross-sectional	5 to 8	Children	383	rDNA	IM	6,10,14 wks
Ayalew (2019)^25^	Ethiopia	Cross-sectional	5 to 9	Children	404	rDNA	IM	6,10,14 wks
Usman (2019)^27^	Ethiopia	Cross-sectional	5 to 8	Children	284	rDNA	IM	6,10,14 wks
Kedir (2019)^28^	Ethiopia	Cross-sectional	5 to 9	Children	380	rDNA	IM	6,10,14 wks
Schoub (2002)^29^	South Africa	Cross-sectional	1 to 6	Children	769	Plasma-derived	IM	6,10,14 wks
Abed (2016)^22^	Egypt	Cross-sectional	6 to 17	Children	600	rDNA	IM	2, 4, 6 mths
El-Sayed (2009)^23^	Egypt	Cross-sectional	5 to 11	Children	200	rDNA	IM	2, 4, 6 mths
Argaw (2020)^26^	Ethiopia	Cross-sectional	5 to 8	Children	451	rDNA	IM	6,10,14 wks

IM: Intramuscular; wks: weeks; mths: months

### Serum anti-HBs level

**Fig. 2:**
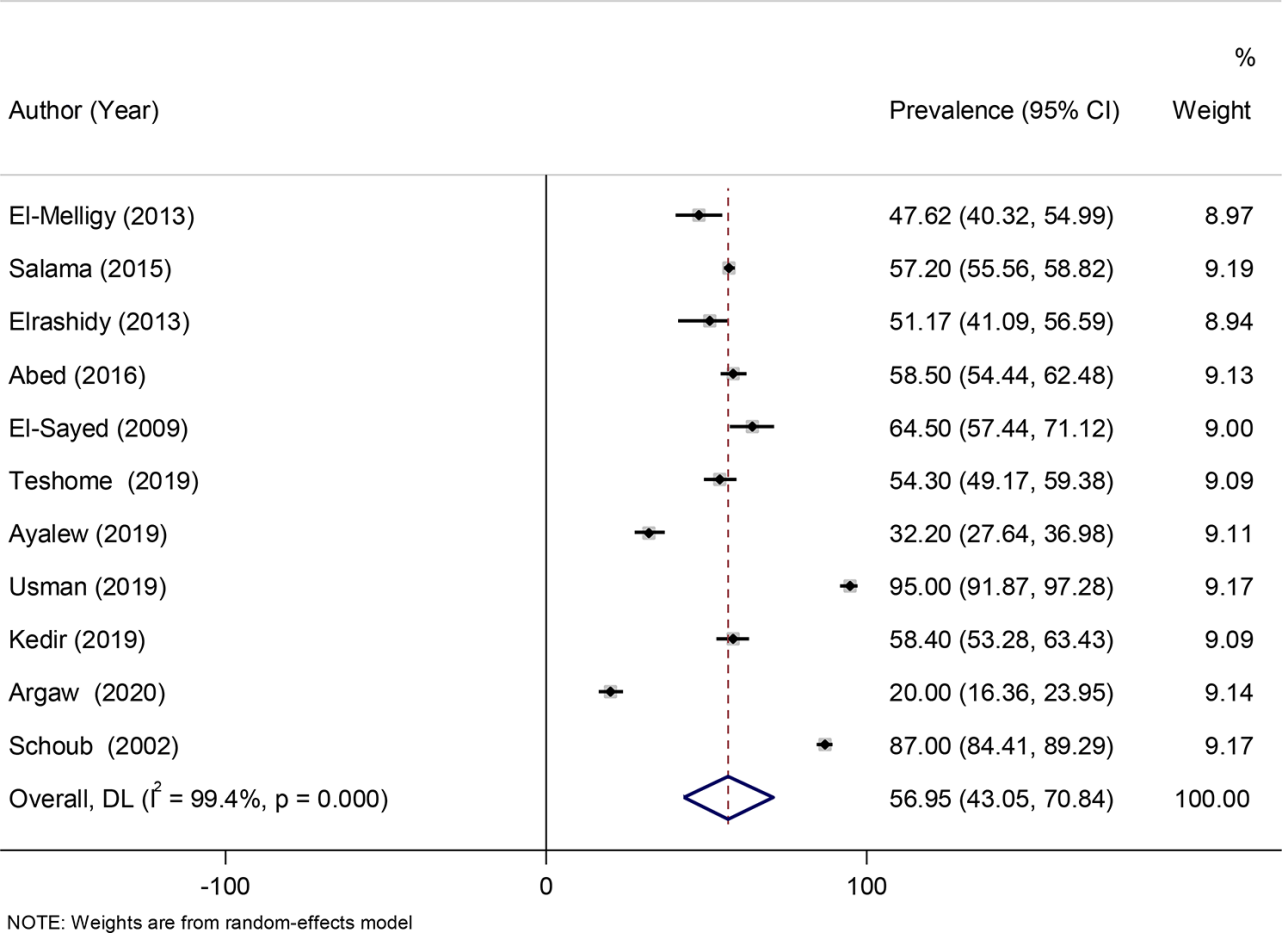
Forest plot of anti-HBs level more than 10mIU/ml

**Fig. 3:**
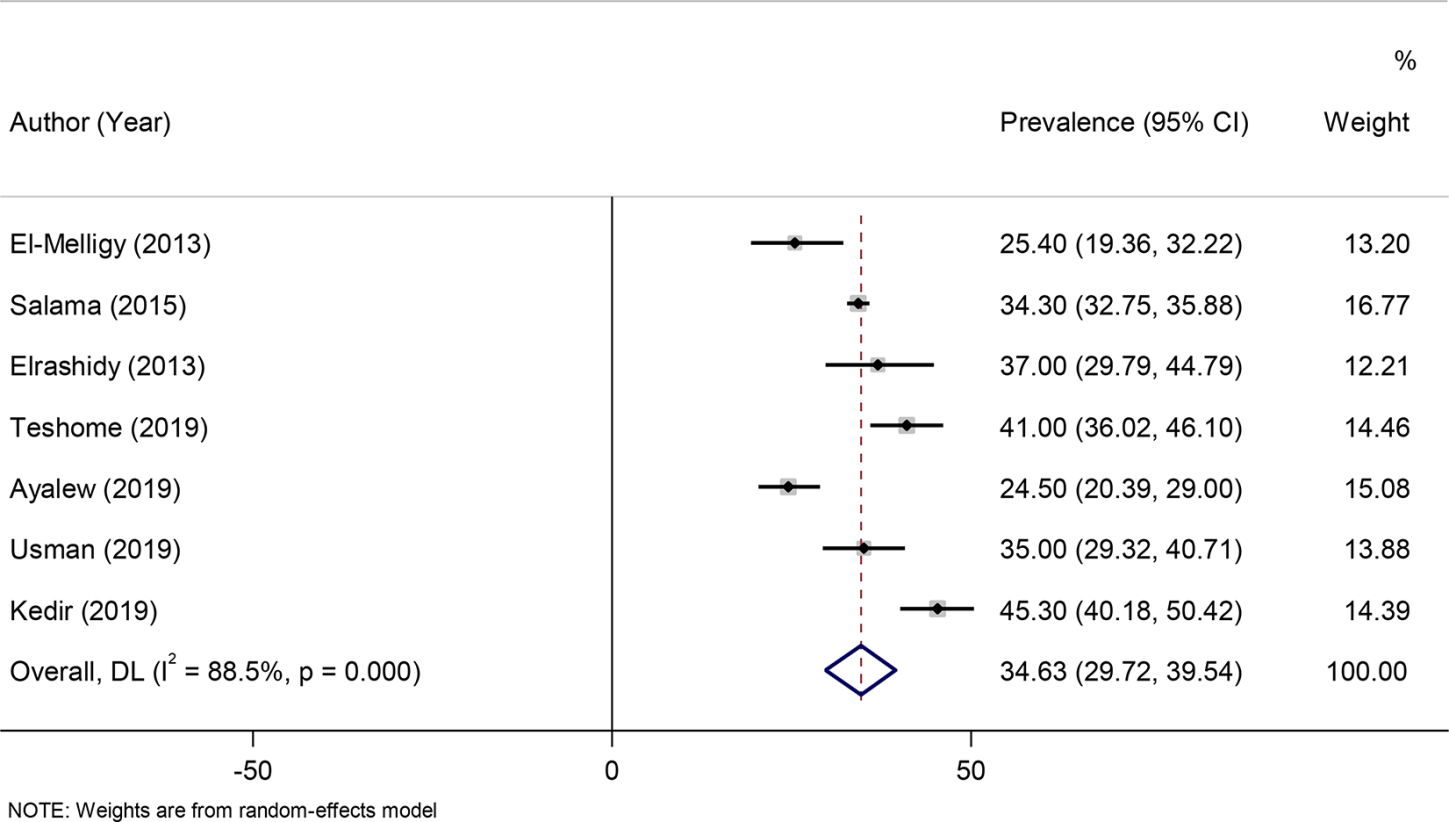
Forest plot of the anti-HBs level between 10 and 99 mIU/ml

**Fig. 4:**
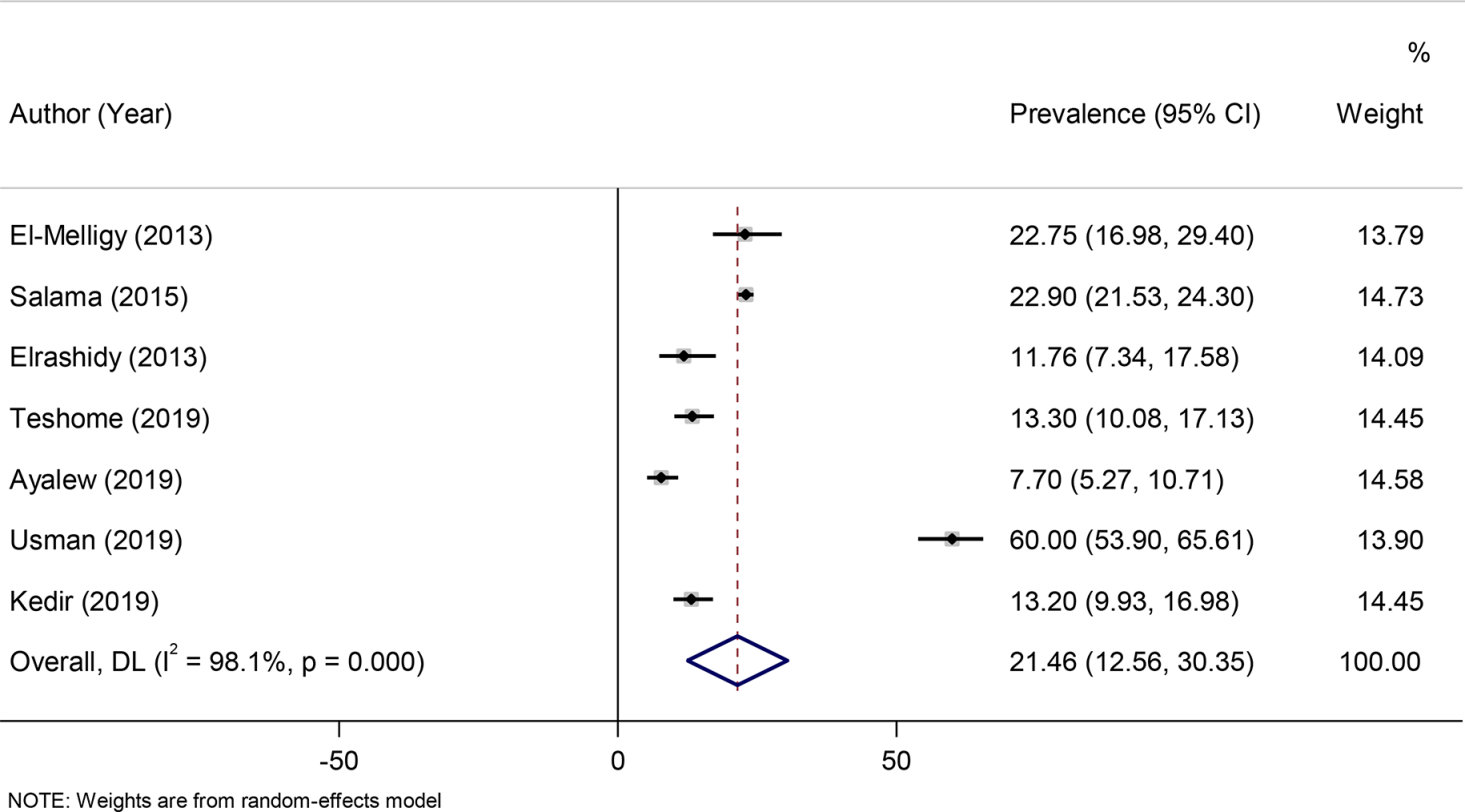
Forest plot of the anti-HBs level more than 100mIU/ml

A random-effects model was fitted to determine the pooled effect size or prevalence of anti-HBs titers. The pooled prevalence of seroprotected children, with anti-HBs titer > 10mIU/ml were 56.95% (95% CI: 43.05–70.84) with a heterogeneity index (I^2^) of 99.4% (*P* < 0.001) (Fig. 2). 35% of the participants (95% CI: 29.72–39.54) were hypo-responder (10-99mIU/ml) (Fig. 3) and 21.46% (95% CI: 12.56–30.35) were hyper responder (> 100mIU/ml) (Fig. 4).

### Heterogeneity and publication bias

**Fig. 5:**
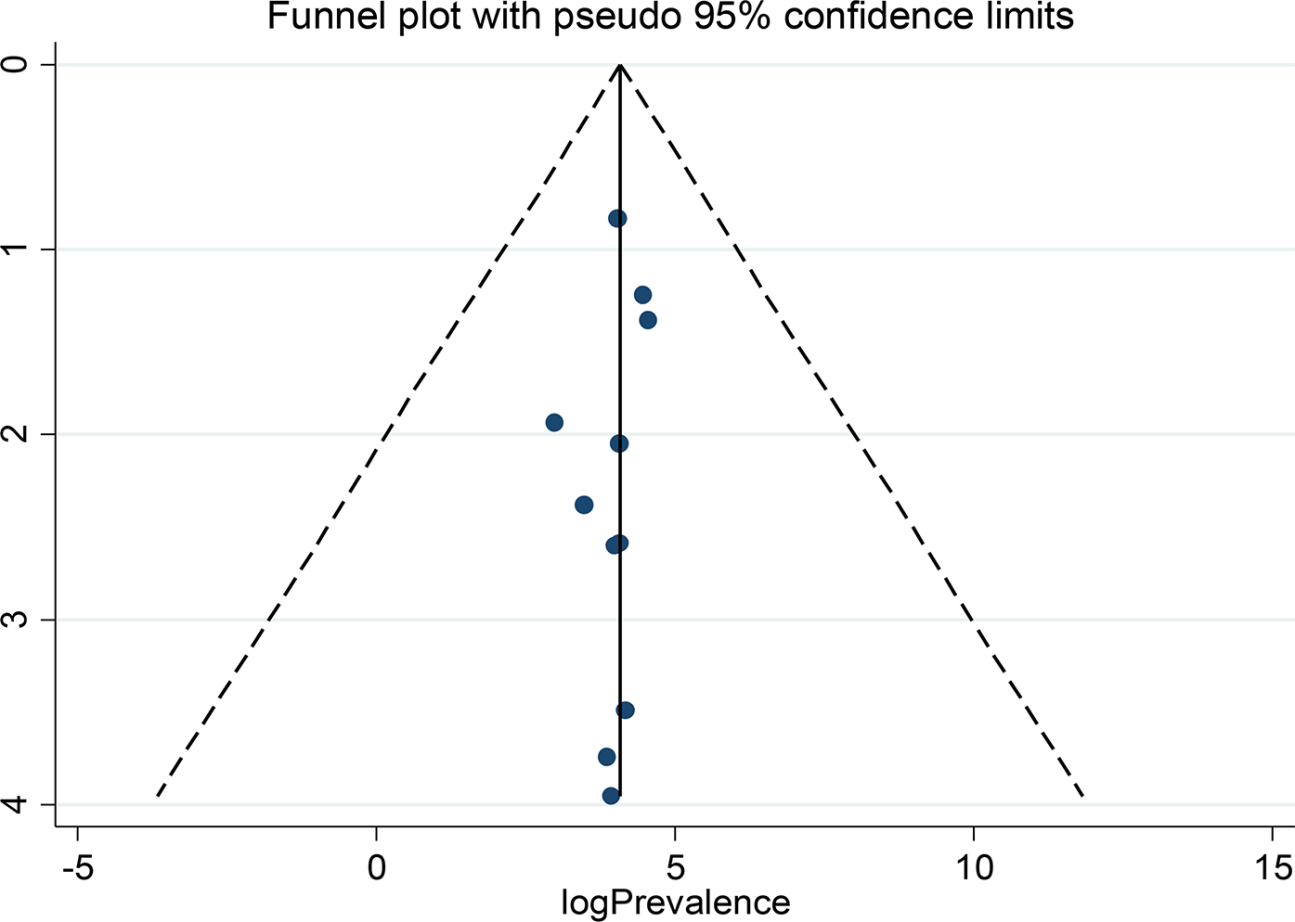
Funnel plot of the anti-HBs level more than 10mIU/ml

The reported heterogeneity of this systematic review and meta-analysis was I^2^ = 98.9%. So, to adjust and minimize, we performed a subgroup analysis based on the country which reported a level of anti-HBs in Africa. Egger test for small-study effects was performed to see the publication bias and graphically by a funnel plot. Visual inspection of the funnel plot indicated symmetrical distribution, which is not statistically significant, as evidenced by the Egger test (*P* = 0.394). The results showed no publication bias (Fig. 5). There was no single study effect in the sensitivity analysis.

### Subgroup analysis

**Fig. 6:**
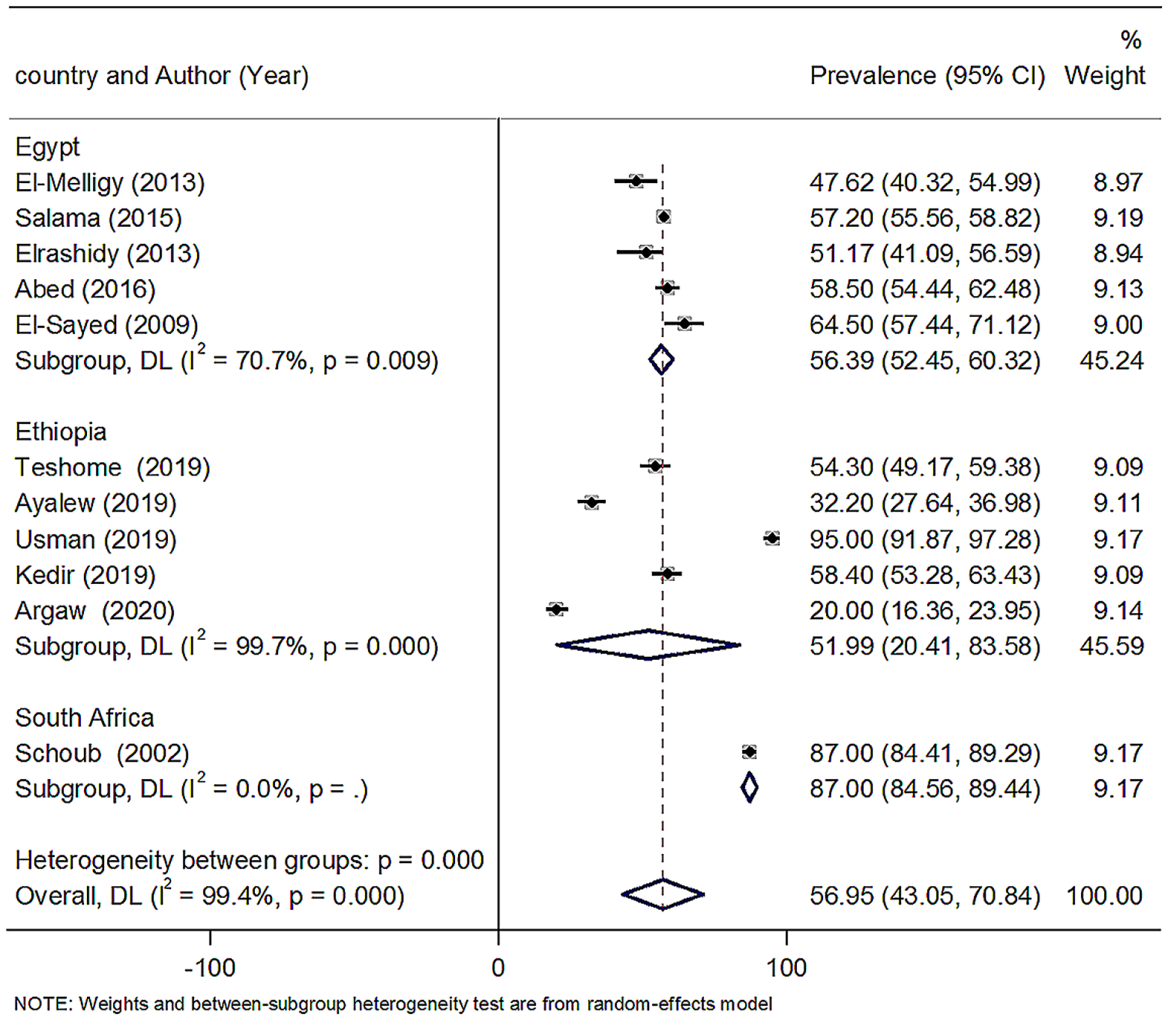
Forest plot of subgroup analysis by country of anti-HBs level more than 10mIU/ml

Since this meta-analysis showed significant heterogeneity, subgroup analysis was done using the country as a subgroup variable. Based on this, the highest anti-HBs level, 87.00% (95% CI: 84.56, 89.44), I^2^ = 0.00% and the lowest (51.99; 95% CI: 20.41–83.58) with a heterogeneity index I^2^ = 70.7% (*p* = 0.009) was observed in South Africa and Ethiopia, respectively. The seroprotective level in Egypt was 56.39 (95% CI:52.45–60.32) (Fig. 6). The highest hypo-responders prevalence of anti-HBs was reported in Ethiopia (36.39%; 95% CI: 26.84–45.95), and the lowest was in Egypt (32.39; 95% CI:26.62–38.16). In addition, the highest good responders prevalence of anti-HBs was reported in Ethiopia (23.36%; 95% CI: 6.50, 40.21), and the lowest was from Egypt (19.28; 95% CI: 12.25, 26.30).

## Discussion

Hepatitis B virus infection and its complications are currently a major issue worldwide. Children are one of the high-risk populations for HBV infection. Neonatal HBV vaccination is the best measure for preventing HBV infection in countries with an intermediate to high HBV endemicity [30]. As recommended by the WHO, all infants should receive the hepatitis B vaccine [31]. A positive immune response to the vaccine is the development of anti-HBs at a titer of > 10 mIU/mL. Based on the vaccine effectiveness studies, it is measured after a complete and adequate vaccination schedule preferably 1 to 3 months of the last vaccination [14, 15, 32].

In the present systematic review, the presence of anti-HBs was used as evidence of the seroprotective level of the HB vaccine. The included studies have used recombinant and plasma-derived HBV vaccines given in different vaccination schedules. Anti-HBs level of more than 100mIU/ml is an essential indicator for good responders, even though not all studies reported it. In contrast, anti-HBs > 10mIU/ml is the most consistent endpoint reported in all of the included studies for assessing the effectiveness of the HBV vaccine. Thus, the vaccine effectiveness measure of the current study considers anti-HBs level > 10mIU/ml as a cutoff point for seroprotectiveness among the vaccinated children.

Therefore, the current systematic review and meta-analysis was conducted to estimate the pooled level of anti-HBs after vaccination among studies conducted in African children. The estimated pooled anti-HBs level was 56.95%. The finding of the vaccines effectiveness in reducing the prevalence of HBV infection, which is 56.95% or less in this review, is suboptimal. It seems unlikely that any country using vaccination to reduce chronic hepatitis B will attain the WHO goal of a 90% reduction of new cases by 2030 [9]. This result was lower than an individual study conducted in South Africa, where the prevalence of anti-HBs after vaccination was 87.0% [29]. This might be due to differences in the standards of health care services, vaccination schedules, type of vaccines and socioeconomic status in various countries.

However, the pooled result was similar to a study conducted in Ethiopia among both vaccinated and unvaccinated children, 65% [33] and a study conducted in Iran, 60.2% [34]. Besides, the present finding showed a higher level of anti-HBs than a study conducted in three African counties, with 42% of children being anti-HBs titer ≥ 10 mIU/mL [35]. This might be because the cold chain systems, different vaccination schedules and unavailability of vaccine might lead the low protectiveness.

Based on the subgroup analysis, the highest seroprotective level were from South Africa, 87.00% Egypt, 56.39%, and the lowest was observed in Ethiopia, 51.99%. The difference might be because of the sample size and the number of included studies in this meta-analysis. For instance, the largest sample size in this study was from Egypt, and the lowest number of studies were from South Africa.

The proportion of individuals with anti-HBs ≥ 10 mIU/mL decreased by age, confirming what has been known for many years [36]. It was observed that serum anti-HBs levels declined when the age increased in studies conducted in different parts of Ethiopia, including Addis Ababa [24], Gondar [25], and Hawassa [26]. It also decreased with the age of the children increased in a study conducted in Egypt [21]. However, the seroprotective level of vaccinated children does not significantly differ among different age groups in a study conducted at Jimma [28].

We have also analyzed the pooled hypo-responders and good responders. The HBV vaccine had a statistically significant effect on hypo-responders and good responders. From the pooled prevalence of seroprotected children in the present meta-analysis, 35% and 21.46% were hypo-responders (10-99mIU/ml) and good responders (> 100mIU/ml), respectively. This revealed higher seroprotected children than in different individual studies conducted in Ethiopia. The first study was conducted at Gondar, reporting that 32.2% had seroprotective titers of anti-HBs > 10 mIU/mL, 24.5% were hypo-responders, and 7.7% were good responders [25]. At Hawassa, 11.6% had HBV immunity with an anti-HBs level > 10 mIU/ml [26]. But our result was lower than a study conducted in Harar, with 95% of the vaccinated children seroprotected with an anti-HBs level > 10mIU/ml. Among these, 35% and 60% of the vaccinated children were hypo-responders and good responders, respectively [27]. Our result was in line with a study conducted at Addis Ababa, which showed as 54.3% had a protective level of antibody [24], and 58.4% of the vaccinated children in Jimma had a protective response to the vaccine with anti-HBs antibody levels ≥ 10 mIU/ml [28].

This meta-analysis result was also in line with a study conducted in Egypt, Alexandria, with 58.5% of the participants were an anti-HBs level > 10 mIU/ml [22], and 47.62% of the vaccinated children responded to the HBV vaccine with an anti-HBs level > 10 mIU/ml [21]. The protective level of children younger than 6 was 95.2%, but 41.7% was in children older than six [21]. It was in line with other study conducted in Egypt, 57.2% showed seroprotective level of anti-HBs > 10mIU/ml, 34.3% and 22.9% were hypo-responders and good responders, respectively [20].

The outcomes of this systematic review might be influenced by clinical heterogeneity, which might occur due to different vaccination schedules, the participants’ age, and the countries’ cold chain systems. Thus, using consistent vaccination schedules and age may have a significant value for the decision of the vaccine effectiveness to have a potent effect on the prevention of HBV infection and CHB. The vaccine effectiveness was also influenced by statistical heterogeneity, but random effects model [36] treated this to remove or reduce the heterogeneity. Although this systematic review and meta-analysis provide up-to-date evidence regarding the HB vaccination seroprotective level, some limitations must be considered in future research. These include the protocol was not registered however Cochrane review was searched and no registered protocol was observed. It includes only papers published in English. The included papers used different age categories which makes difficult for subgroup analysis to a factor for seroprotective the current meta-analysis.

In conclusion, HB vaccine seroprotective level in the current pooled analysis have suboptimal, which failed to demonstrate consistent effectiveness for global hepatitis B virus elimination plan in 2030. Using consistent age group may have a significant value for the decision of the HB vaccine effectiveness. A significant heterogeneity was observed both in studies conducted in Ethiopia and Egypt. Therefore, the impact of HB vaccination on the prevention of hepatitis B virus infection should be assessed regularly in those countries. Future meta-analysis is needed to investigate all possible vaccines in a separate way of reviewing, which will lead to a strong conclusion and recommendations.

### Electronic supplementary material

Below is the link to the electronic supplementary material.

**Supplementary Material 1:** PRISMA 2009 Checklist

**Supplementary Material 2:** NEWCASTLE – OTTAWA QUALITY ASSESSMENT SCALE

## Data Availability

The data used for this systematic review are available at the corresponding author, so that interested reader can get the data from the corresponding author with reasonable request.

## Abbreviations

Anti-HBs: Hepatitis B Surface Antibody
CHB: Chronic Hepatitis B
CI: Confidence Interval
HBV: Hepatitis B Virus
mIU: Milli-International Unit
ml: Milliliter
PRISMA: Preferred Reporting Items for Systematic Reviews and Meta-Analyses
STATA: Statistical Software for Data Science
WHO: World Health Organization
